# Chemopreventive effects of angiotensin II receptor type 2 agonist on prostate carcinogenesis by the down-regulation of the androgen receptor

**DOI:** 10.18632/oncotarget.24492

**Published:** 2018-02-14

**Authors:** Yusuke Ito, Aya Naiki-Ito, Hiroyuki Kato, Shugo Suzuki, Toshiya Kuno, Yukari Ishiguro, Satoru Takahashi, Hiroji Uemura

**Affiliations:** ^1^ Department of Urology, Yokohama City University Graduate School of Medicine, Yokohama, Japan; ^2^ Department of Experimental Pathology and Tumor Biology, Nagoya City University Graduate School of Medical Sciences, Nagoya, Japan; ^3^ Department of Urology and Renal Transplantation, Yokohama City University Medical Center, Yokohama, Japan

**Keywords:** prostate cancer, RAS, angiotensin II receptor type 2, compound 21

## Abstract

We recently reported that angiotensin II receptor blockers (ARBs) have chemopreventive and chemotherapeutic potential against prostate cancer via the reduction of androgen receptor (AR) expression. In this study, we investigated the effects of the angiotensin II receptor type 2 (AT2R) agonist Compound 21 (C21), which is expected to play similar roles to an ARB, on prostate carcinogenesis using the transgenic rat for adenocarcinoma of prostate (TRAP) model previously established in our laboratory. *In vitro* analyses of the cell growth, Western blotting and reporter gene assays were performed using LNCaP cells. TRAP rats at 6 weeks of age were randomly divided into 3 groups of 12 animals each and treated with C21 at 1 or 2 mg/kg/day in drinking water for 12 weeks. C21 reduced the proliferation activity of prostate cancer cells and down-regulated the PSA promoter activity and the AR protein expression. We discovered that C21 inhibited the progression of prostate carcinogenesis in TRAP rats and decreased the incidence of adenocarcinoma in the lateral prostate. A significant increase in the apoptotic index with activation of caspase 3 and 7 were observed by immunohistochemistry and Western blotting analyses. C21 also down-regulated the expression of AR significantly in TRAP rat prostate. C21 decreased the expression of AR and reduced the proliferation activity effectively in prostate cancer cells and TRAP rat prostate. These findings suggest that AT2R agonist may be a candidate novel chemopreventive agent against human prostate cancer.

## INTRODUCTION

Prostate cancer is the most common cancer in men in the United States. More new cases of prostate cancer were reported than for any other cancer in men, and prostate cancer was the third leading cause of death from cancer in 2017 [1]. The incidence of prostate cancer is also increasing in Asia. The carcinogenic process in the prostate gland is initially androgen-dependent, so the basic therapeutic strategy has been the androgen ablation [2]. However, despite an initial clinical response, the progression to castration-resistant disease is nearly universal, posing a serious problem for the outcome of prostate cancer. Consequently, there is a need to identify new chemopreventive or therapeutic strategies for prostate cancer.

Previous experiments by our groups have shown that angiotensin II type 1 receptor (AT1R) blockers (ARBs) have anti-proliferative activity in prostate cancer cells and have a therapeutic effect clinically [3–10]. It is generally known that angiotensin II binds to two kinds of receptors, AT1R and AT2R, and the biological function of AT2R signaling has the opposite effect to that of AT1R signaling in many aspects [11, 12]. We therefore hypothesized that AT2R agonism might attenuate the proliferative activity in prostate cancer cells. For a long time, no appropriate compound for the investigation of the AT2R function existed, due to the low selectivity. Now, however, we use the highly selective cutting edge agonist Compound 21 (C21) designed by Vicore Pharma (Sweden).

We previously established a transgenic rat for adenocarcinoma of prostate (TRAP) model, which harbors a transgene encoded simian virus (SV40) T antigen under the probasin promoter [13]. The TRAP model develop androgen-dependent neoplastic lesions, and prostatic intraepithelial neoplasia (PIN) and adenocarcinoma *in situ* were evident at 15 weeks of age. With further aging or intermittent testosterone propionate administration, invasive adenocarcinoma was observed in the prostate of the TRAP model [14, 15]. Therefore, the TRAP model may be a good tool for evaluating an agent’s chemopreventive action in prostate carcinogenesis in a short period. Using this transgenic rat model, the chemopreventive effects of several chemicals and drugs, such as gamma-tocopherol, apocynin, ellagic acid, and ARBs, have been established [5, 16–18].

In this study, we examined the chemopreventive effects of the AT2R agonist C21 on prostate carcinogenesis and investigated whether or not C21 down-regulates androgen receptor (AR) or induces apoptosis, as with ARBs.

## RESULTS

### C21 reduced the proliferation activity of prostate cancer cells and down-regulated the expression AR

**Figure 1:**
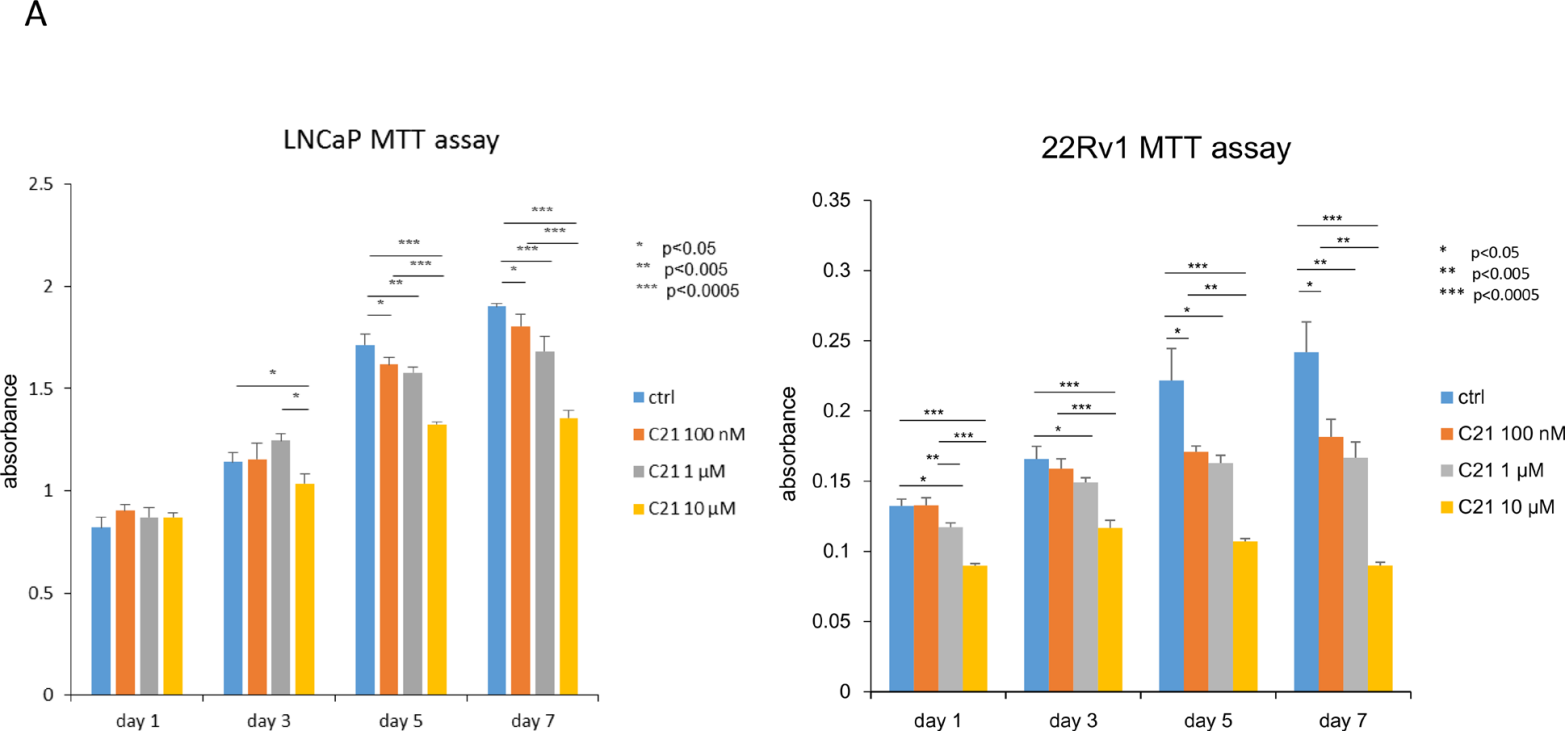

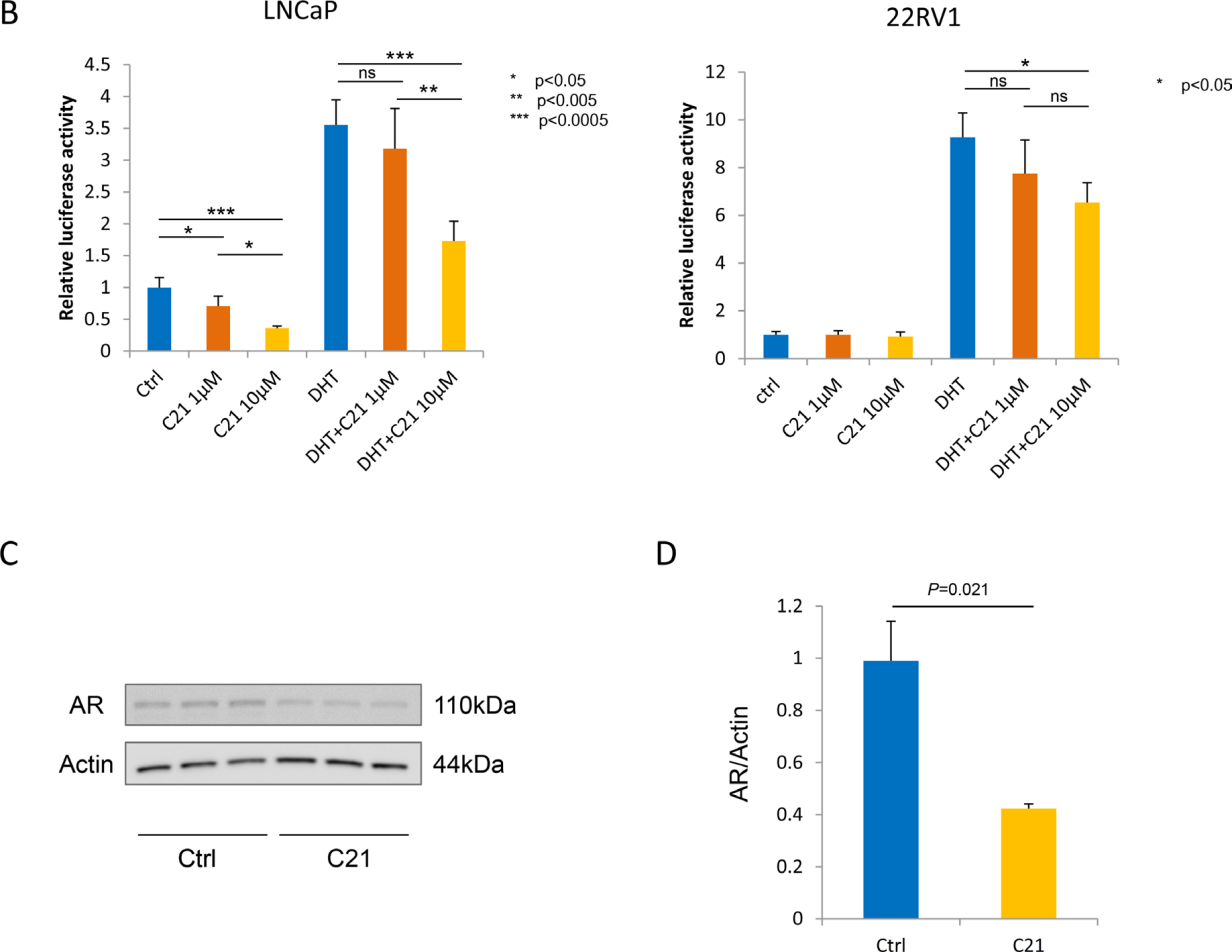
C21 suppressed cell proliferation and down-regulated the expression of AR in LNCaP and 22RV1 cells (**A**) An MTT assay was performed, and the cells were counted after daily treatment with 100 nM, 1 or 10 µM of C21. LNCaP or 22RV1, 3 × 10^3^ cells/well were seeded in a 96-well plate, and all experiments were repeated 3 times. Data are presented as means ± SD, *n* = 6 per group, ^*^P < 0.05, ^**^P < 0.005, ^***^P < 0.0005 vs. the control group. (**B**) A luciferase reporter assay was performed. LNCaP and 22RV1 cells, 10^3^ cells/well were seeded in a 24-well plate. The cells were treated with 1 or 10 µM of C21 with or without 1 nM of DHT, and all experiments were repeated 3 times. Data are presented as means ± SD, ^*^P < 0.05, ^**^P < 0.005, ^***^P < 0.0005 vs. the control group. (**C**) A Western blot assay of AR and β-actin in LNCaP cells. LNCaP cells were incubated with 10 µM of C21. The data are representative of three independent experiments. (**D**) The intensity of the Western blot band was measured using the ImageJ software program and normalized to β-actin.

First, we determined whether or not C21 down-regulated the growth of prostate cancer cells in an MTT assay. As shown in Figure 1A, LNCaP and 22RV1 cells treated with C21 in complete medium for up to 7 days showed a significant, dose-dependent decrease in cell growth compared to controls. We then examined the relationship between C21 and AR using a PSA-promoter luciferase reporter assay. As shown in Figure 1B, after 3 days’ incubation, C21 at 10 µM had significantly decreased the transcriptional activity with or without 1 nM of dihydrotestosterone (DHT) stimulation in LNCaP cells to control or DHT alone treated cells (*P* < 0.05, *P* < 0.005, and *P* < 0.0005, respectively). In addition, C21 at 10 µM showed a significant decrease in the luciferase activity with 1 nM of DHT stimulation in 22RV1 cells (*P* < 0.05). To confirm whether or not C21 could reduce the PSA level, we examined the PSA in LNCaP cells treated with C21 plus DHT. As expected, the PSA levels decreased after C21 stimulation (supplemental data in Supplementary Figure 1). To assess the expression of AR, we conducted a Western blot analysis in LNCaP cells after three consecutive days’ stimulation with 10 µM C21. C21 reduced the protein levels of AR to less than half of that in controls (*P* = 0.021) (Figure 1C and 1D). These results suggest that C21 has the potential to down-regulate the expression of AR and its transcriptional activity, and accordingly, to reduce the proliferation activity in prostate cancer cells.

### Suppressive effects of AT2R agonist on prostate carcinogenesis in TRAP via the induction of caspase-dependent apoptosis

**Figure 2:**
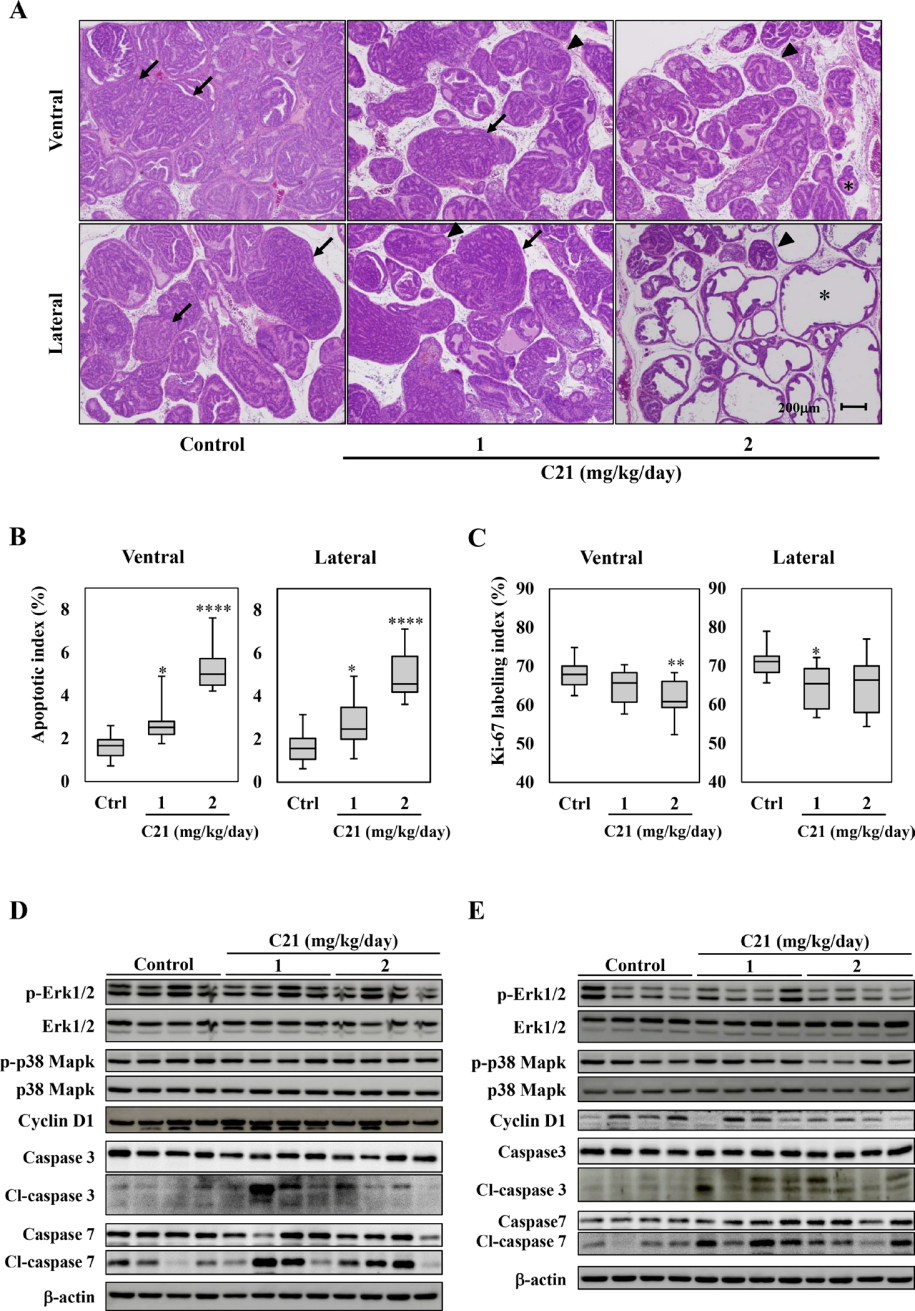
AT2R agonist supplementation suppressed the progression of prostate carcinogenesis and induced apoptosis in the TRAP model (**A**) Representative histological findings of VP and LP of TRAP. Representative histological findings of adenocarcinoma (arrow), HG-PIN (arrowhead), and LG-PIN (asterisk). (**B**, **C**) Labeling indices for Ki67- (B) and TUNEL- (C) positive cells in VP and LP. Hematoxylin was used as a nuclear counterstain. Data are presented in a box plot, *n* = 12 per group, ^*^P < 0.05, ^**^P < 0.01, ^****^P < 0.0001 vs. the control group. (**D**, **E**) A Western blotting analysis for Erk 1/2, phospho-Erk 1/2, p38 Mapk, phosphor-p38 Mapk, Cyclin D1, caspase 3 and 7, cleaved (cl)-caspase 3 and 7, and β-actin in VP (D) and LP (E) of TRAP. Western blotting was repeated at least three times, and each lane represents an individual rat (*n* = 4 per group).

The blood pressure was significantly reduced in TRAP rats treated with C21 compared to controls (Supplementary Table 1). The administration of C21 in drinking water did not affect the body weight or the weights of the liver, kidneys, or VP, and there were no significant changes in serum testosterone levels (Supplementary Tables 1 and 2). The estradiol level in serum showed a dose-dependent increase in TRAP with C21; however, there was no significant difference in the T/E2 ratio among the groups (1, 2 mg/kg/day; *P* = 0.31, 0.37 vs. control group). A histological analysis revealed that C21 treatment significantly suppressed the progression of prostatic lesions from LG-PIN to HG-PIN or adenocarcinoma in both VP and LP of TRAP (Table 1 and Figure 2A). In VP, the percentage of LG-PIN was significantly increased by both doses of C21, and the percentage of adenocarcinoma was significantly decreased by the high dose. As in LP, the percentage of LG-PIN was higher and adenocarcinoma lower in TRAP rats with a high dose of C21 compared to the control group. Furthermore, incidence of adenocarcinoma was significantly decreased by high dose of C21 treatment in LP as compared to control.

**Table 1:** Incidence of carcinoma and quantitative evaluation of neoplastic lesions in prostates of TRAP rats treated C21

	No. of rat	Ventral				Lateral			
		Incidence of carcinoma	% of lesions in prostate			Incidence of carcinoma	% of lesions in prostate		
			LG-PIN	HG-PIN	Adenocarcinoma		LG-PIN	HG-PIN	Adenocarcinoma
Control	12	12 (100%)	6.9 ± 2.5	82.9 ± 5.4	10.3 ± 4.4	12 (100%)	12.0 ± 11.0	79.6 ± 10.9	8.4 ± 4.0
C21 1 mg/kg/day	12	12 (100%)	16.7 ± 4.8^****^	73.9 ± 4.6	9.4 ± 3.9	10 (83%)	12.6 ± 9.4	79.5 ± 13.1	7.9 ± 7.4
C21 2 mg/kg/day	12	12 (100%)	14.1 ± 3.1^***^	80.8 ± 3.9	5.2 ± 4.0^*^	7 (58%)^*^	20.2 ± 11.0^***^	77.0 ± 9.7	2.8 ± 4.0^*^

^*^p < 0.05, ^***^p < 0.001, ^****^p < 0.001: significantly different from control group.

To confirm the effects of C21 on cell proliferation or apoptosis during prostate carcinogenesis, the labeling indices of Ki-67 and TUNEL in HG-PIN among each group were evaluated. Apoptotic indices were significantly increased in a dose-dependent manner in the VP and LP of TRAP rats treated with C21 as compared to control (Figure 2B and Supplementary Figure 2A). The Ki-67 labeling indices were slightly decreased by C21 in both VP and LP (Figure 2C and Supplementary Figure 2B). Western blotting analyses showed the activation of caspase 3 and 7 in VP and LP of TRAP treated with C21, while the Erk1/2, p38 Mapk activity and cyclin D1 expression was not altered by the treatment (Figure 2D and 2E).

### Down-regulation of AR signaling by AT2R agonist in TRAP rats

**Figure 3:**
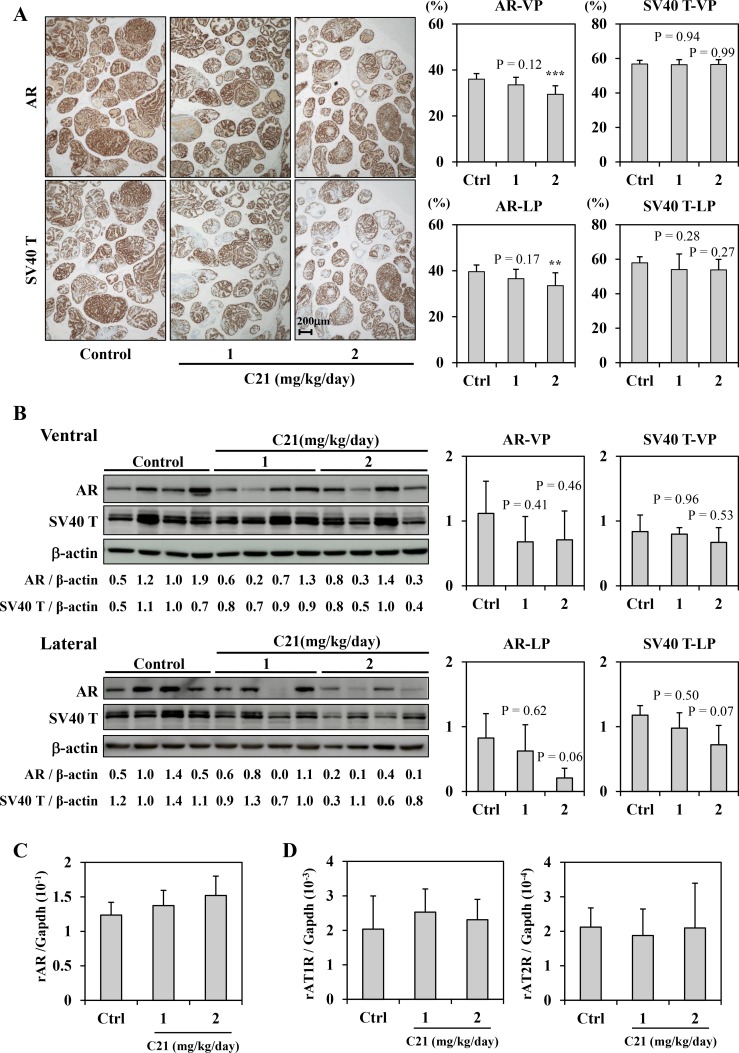

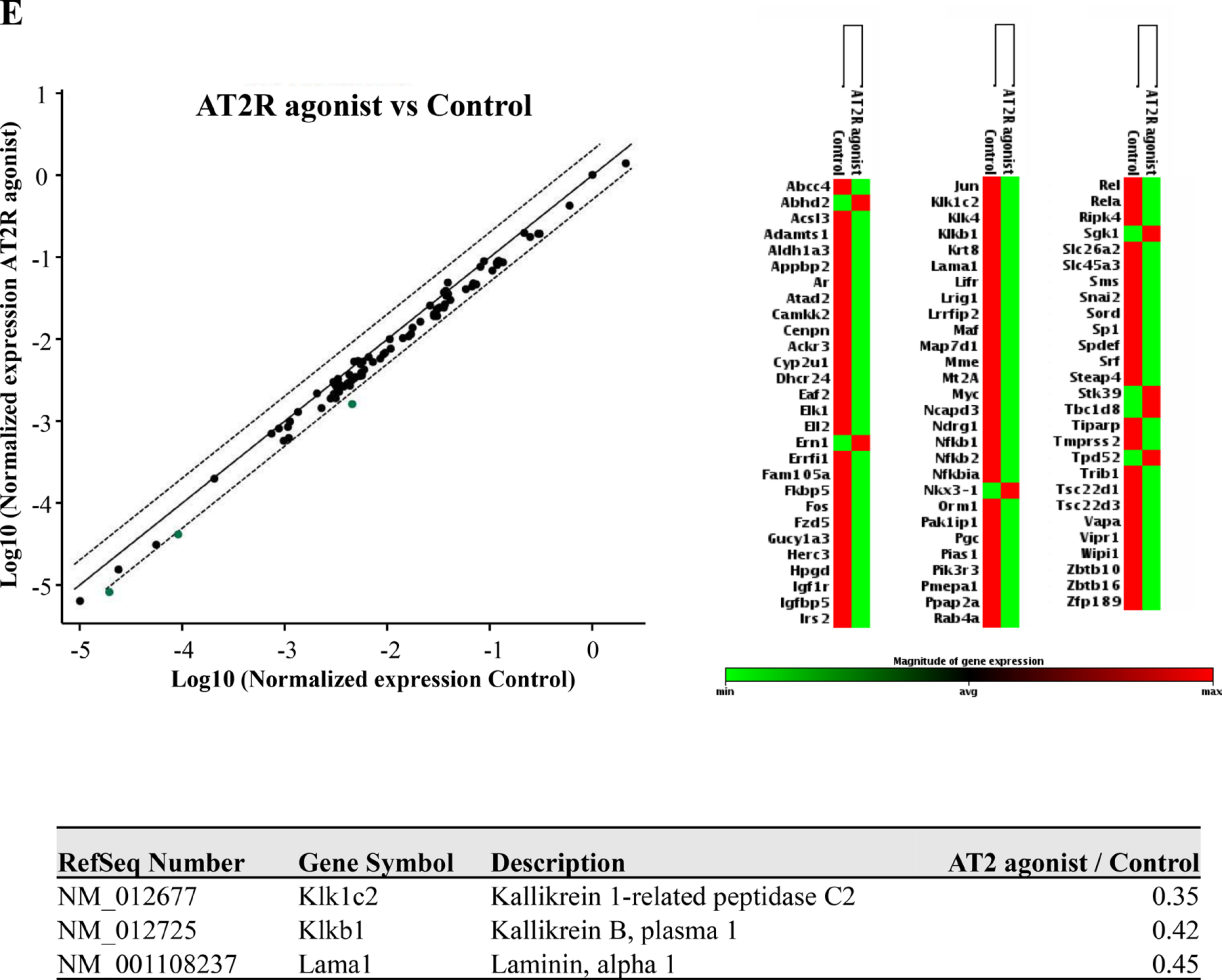
AT2R agonist down-regulated AR signaling (**A**) Immunohistochemical staining for AR and SV40 T antigen in the prostate lobes of TRAP rats. Representative photographs for each staining of LP (left). Hematoxylin was used as a nuclear counterstain. The percentages of positive cells for each staining (right). Data are presented as the mean ± SD, *n* = 12 per group, ^**^P < 0.01, ^***^P < 0.001 vs. the control group. (**B**) A Western blotting analysis for AR, SV40 T antigen, and β-actin in VP and LP of TRAP. The intensity of each band was measured and normalized by β-actin. Western blotting was repeated at least three times, and each lane represents an individual rat. Data are presented the relative value for a Control (Ventral: lane 3, Lateral: lane 2) as the mean ± SD, *n* = 4 per group. (**C**, **D**) A quantitative RT-PCR analysis for AR (C), AT1R, and AT2R (D) in LP of TRAP rats. The average results were calculated from five rats per group. Each PCR was repeated 4 times. The data was normalized with Gapdh as an endogenous control gene. (**E**) RT-PCR array analysis of the effect of C21 on the expression of 84 androgen-regulated genes in LP of TRAP rats. RNA samples from all rats in the Control (*n* = 12) and the 2 mg/kg/day C21 (*n* = 12) groups were analyzed. The data was normalized to the Actb gene. Top left, scatter plot: Green dots represent genes that were down-regulated. The center line indicates no changed in gene expression, while the boundary dotted lines represent a 2-fold change in expression. Top right, clustergram: non-supervised hierarchical clustering of the entire data set to display a heat map indicating gene alteration across groups (83 out of every 84 genes). Red shows relatively high expression level (*n* = 7), green shows relatively low expression level (*n* = 76) as compared with the other group. Bottom, List of genes down-regulated more than double by C21 treatment.

Since ARBs suppressed AR expression in TRAP rats and the human prostate cancer cell line LNCaP in our previous study [5], we next investigated the effect of C21 on AR expression in TRAP rats. Immunohistochemical analysis of prostate tumors of TRAP rats showed that the localization of AR and SV40 T expression was not altered in C21-treated animals, but did reveal a significant dose-dependent decrease in AR expression in both the VP and LP (Figure 3A). Western blotting confirmed a similar dose-dependent decrease in AR expression in the LP (Figure 3B). In the VP, AR expression appeared to be decreased with C21 treatment, but this change was not statistically significant (Figure 3B). In contrast, there was no marked difference in the AR mRNA expression among the groups (Figure 3C). The protein expression of SV40 T was much reduced in LP, although there was no significant difference among the groups (Figure 3A and 3B). The expression of AT1R and AT2R mRNA was not affected by C21 (Figure 3D).

To further clarify the suppressive effect of C21 on AR, the mRNA expression of AR responsive genes in LP was compared between the control and C21 high-dose group by a reverse transcription polymerase chain reaction (RT-PCR) array. Among 85 genes, the expression of 76 was lower in the LP of TRAP rats treated with C21 than in controls (Figure 3E). In particular, kallikrein 1-related peptidase C2 (Klk1c2), kallikrein B, plasma 1 (Kikb1), and laminin, alpha 1 (Lama1) were down-regulated by C21 treatment (ratio of C21 to control: 0.35, 0.42 and 0.45, respectively) (Figure 3E).

## DISCUSSION

With the emergence of new agents, such as enzalutamide and abiraterone, a paradigm shift is occurring in prostate cancer treatment, especially for castration-resistant prostate cancer (CRPC). Those new agents clarified that even in the CRPC stage, AR continues to work as a key molecule. However, the effect is limiting, and prostate cancer circumvents these agents in various ways, including via AR variants and the *de novo* production of androgen by itself [19]. Although several new drugs are under development, including EPI-002, Galeterone, and ARN-509 [20, 21], we must find other ways to maintain control of AR.

In previous studies, we presented evidence that ARBs decrease PSA in the clinical setting and suppress prostate cancer proliferation through experimental AT1R blockade [3–10, 22]. Angiotensin II can bind to two main receptors (AT1R and AT2R), and the blockade of AT1R is known to result in the direct inhibition of the AT1R function that induces cell proliferation via signal transduction. However, AT2R agonism in prostate cancer had not previously been examined, due to a lack of proper AT2R agonists.

In the present study, we investigated the agonistic effect of AT2R in prostate cancer using C21, which was developed as a new, orally active, high-affinity AT2R agonist [23]. C21 has a broad therapeutic platform, including cardiovascular, anti-inflammation, anti-fibrosis, and neuroprotective effects. Unlike AT1R, AT2R is expressed ubiquitously in the fetus but is organ-specific in adults. In addition to expression in the adrenal medulla, brain, and ovarian follicles, several studies have demonstrated that the prostate also expresses AT2R [24, 25]. Our examination of whether or not C21 affected the expression of AT1R in prostate cancer cells revealed that C21 did not markedly affect the AT1R expression in LNCaP cells (data not shown) or *in vivo* (as shown in Figure 3D). Because it is commonly accepted that AT2R and AT1R work in a contradictory manner [11,12], we hypothesized that AT2R agonism suppresses the proliferation activity in prostate cancer cells and down-regulates AR, similar to ARBs. As expected, we confirmed that C21 treatment suppressed the proliferation activity and down-regulated the expression of AR in prostate cancer in both *in vitro* and *in vivo* experiments.

Previous studies have suggested that prostate cancer shows low expression of AT2R compared to noncancerous prostate tissue, and the expression is reduced according to the escalation of the Gleason score [24, 25]. However, the low expression of the receptor does not exclude the possibility that the stimulation of the receptor induces a great effect. In fact, C21 has high-affinity for AT2R (*K*_*i*_ value = 0.4 nM) compared to AT1R (*K*_*i*_ value > 10 µM) [23], and the stimulation of AT2R by C21 showed a dramatic effect in our study. Li *et al.* reported that the over-expression of AT2R in prostate cancer cells induced cell apoptosis [25]. Our study also showed the induction of apoptosis by caspase 3/7 cleavage and a TUNEL assay, findings that coincide with those of previous reports [25, 26].

C21 reduced the expression of AR and significantly decreased the PSA luciferase activity *in vitro*. As shown in Figure 1A and 1B, C21 decreased the cell proliferation of LNCaP and 22RV1 because of the down-regulation of the AR expression. C21 supplementation also decreased the AR expression and down-regulated AR-responsive genes in the prostate of the TRAP model (Figure 3E). However, the AR mRNA level was not markedly changed *in vivo*. Although the precise mechanism by which C21 reduced the expression of AR has not been fully elucidated, these findings imply that C21 affects post-translational changes of the AR. A previous study found that ARBs suppressed AR protein expression suggested that the mechanism involved a proteasome-dependent pathway mechanism [5]. More detailed studies are needed to clarify the post-translational changes in the AR induced by C21 or via AT2R stimulation.

Since the SV40 T antigen expression is controlled by the probasin promoter, which is an androgen-responsive gene, prostate carcinogenesis in TRAP rats is thought to be affected by chemicals modulating the AR axis. The expression of SV40 T in the lateral lobe was considerably decreased, but not to a significant degree, in the present study, so we cannot exclude the possibility that the suppressive effects of C21 may have been due to SV40 T down-regulation. However, despite a slight decrease in the SV40 T expression in the ventral lobe, prostate carcinogenesis was suppressed to the same level as that of the lateral lobe. Given these data, we suspect that the suppression of prostate carcinogenesis by C21 was not due to the down-regulation of transgene expression.

One limitation of this study is that we only demonstrated the chemopreventive effect of C21 in an *in vivo* model. To clarify the utility of C21 as a treatment drug in prostate cancer patients, we must perform a preclinical study to investigate the effect using a cancer xenograft model. Likewise, ARBs have practically shown PSA reduction to some extent, even in CRPC patients, so C21 might also have a similar anti-cancer effect.

In conclusion, the present study demonstrated that the epoch-making AT2R agonist C21 down-regulated the proliferation activity and the expression of AR both *in vitro* and *in vivo*. To our knowledge, this is the first report of C21 being used in a transgenic rat model that spontaneously develops cancer. C21 is a promising drug not only for hypertension but also for human prostate cancer chemoprevention.

## MATERIALS AND METHODS

### Chemicals

The AT2R agonist C21 was provided by Vicore Pharma AB (Gothenburg, Sweden).

### Prostate cancer cell lines

LNCaP and 22RV1 cells were obtained from the American Tissue Culture Collection (Rockville, MD, USA). Each cell was maintained in RPMI (for LNCaP) or phenol-red free RPMI (for 22RV1) medium supplemented with 10% heat-activated fetal bovine serum (FBS) under 5% CO_2_ at 37° C.

### Cell growth

Cell growth was measured by an MTT assay. For the MTT assay, LNCaP and 22RV1, 3 × 10^3^ cells/well were seeded in 96-well plates. C21 was added daily with replenishment in 22RV1 and without it in LNCaP cells. After incubation, 10 µL TetraColorOne (Seikagakukogyo, Tokyo, Japan) was added to each well, and the absorbance was quantified according to the manufacturer’s protocol.

### Luciferase reporter assay

Firefly Luciferase test reporter genes were used in this study. pGL3-PSA, the Luciferase reporter plasmid-driven 6.0-kb prostate antigen (PSA) promoter, was provided by Dr. Chawnshang Chang (Rochester University), and phRL-SV40 as an internal control was obtained from Promega (Madison, WI, USA). Each reporter construct was transfected into prostate cancer cell lines in 24-well plates using Lipofectamine 3000 (Invitrogen, Carlsbad, CA, USA). Cells were incubated for 48 h after transfection. Luciferase activity was measured using Dual-Luciferase Reporter Assay System (Promega) and a plate reader (Infinite 200 Pro; TECAN, Männedorf, Switzerland). The Firefly Luciferase activity was normalized to the Renilla luciferase activity. Six wells of each sample were used, and all experiments were repeated three times. LNCaP and 22RV1 cells were stimulated with 1 or 10 µM of C21 with or without 1 nM of dihydrotestosterone (DHT) (Sigma-Aldrich, St. Louis, MO, USA), and all experiments were repeated three times.

### Western blotting analyses for *in vitro* samples

LNCaP cells treated with C21 were washed in ice-cold PBS and then dissolved in RIPA buffer (R0278; Sigma-Aldrich) with protease inhibitor cocktail (P8340; Sigma-Aldrich). The lysate was centrifuged for 30 min at 15,000 × g at 4° C, and the supernatant was harvested as samples. After the determination of the protein concentration using the Bio-Rad protein assay (Bio-Rad, Hercules, CA, USA), 20 µg of the total cell lysate was electrophoresed by sodium dodecyl sulfate-polyacrylamide gel electrophoresis (SDS-PAGE) and electrophoretically transferred to PVDF membranes using the Trans-Blot^®^ Turbo™ Transfer System (Bio-Rad). iBind™ Western Systems (Thermo Fisher Scientific, Rockford, IL, USA ) were used for blocking and primary and secondary antibody incubation. The primary antibody used in the experiments was AR (N-20) from Santa Cruz Biotechnology (Santa Cruz, CA, USA). β-actin was from Sigma-Aldrich. Specific signals were detected using the ECL Kit (GE Healthcare, Tokyo) and the LAS 4000 imaging system.

### Animals

Heterozygous male TRAP rats used in this study were established in our laboratory with a Sprague-Dawley genetic background, as described previously [13]. All experimental rats were housed three per plastic cage on wood-chip bedding in an air-conditioned specific-pathogen-free (SPF) animal room at 22 ± 2° C and 55% ± 5% humidity with a 12-h light/dark cycle. Food and tap water were available *ad libitum*.

### Experimental protocol

A total of 36 male TRAP rats at 3 weeks of age were randomly divided into 3 groups. Rats in the control group (*n* = 12) received basal diet and tap water. The rats in the other two groups continuously received either 1 or 2 mg/kg/day C21 in drinking water for 12 weeks.

Blood pressure was measured at weeks 0, 4, and 6, and all rats were sacrificed under deep anesthesia at the end of week 12. The prostate was removed from each animal, and half of the ventral prostate (VP) and lateral prostate (LP) lobes were immediately frozen in liquid nitrogen, while the remainder of the prostate was fixed in 10% phosphate-buffered formalin. After formalin fixation for 48 h, the seminal vesicle and remaining VP and LP were trimmed and routinely embedded in paraffin for a histopathological evaluation and immunohistochemistry. Testosterone and estrogen levels in the serum were analyzed by radioimmunoassay by The Tohkai Cytopathology Institute: Cancer Research and Prevention (TCI-CaRP, Gifu, Japan). The present experiments were performed under protocols approved by the Institutional Animal Care and Use Committee of Nagoya City University School of Medical Sciences.

### Assessment of prostate neoplastic lesion development

Neoplastic lesions of the prostate glands were classified as low-grade prostatic intraepithelial neoplasia (LG-PIN), high-grade PIN (HG-PIN), and non-invasive adenocarcinoma, as previously described [16, 27]. The number of LG-PIN, HG-PIN, and adenocarcinoma lesions in the VP and LP was scored blindly by two experts in diagnostic pathology (AN and ST) and presented as a percentage of lesions in each prostate.

### Western blotting analyses for *in vivo* samples

The frozen prostate tissues were homogenized with T-PER Tissue Protein Extraction Reagent (Thermo Scientific) containing a protease inhibitor (Thermo Scientific). Proteins concentrations were quantified by the Bradford procedure and equal amounts of proteins. Samples were loaded at 30 µg per lane, separated on 12% acrylamide gels, and electroblotted onto nitrocellulose membranes (Hybond-ECL; GE Healthcare UK Ltd., Buckinghamshire, UK). The primary antibodies used in this study were caspase 3, caspase 7, cleaved (cl)-caspase 3, cl-caspase 7, Erk 1/2, phospho-Erk 1/2, p38 Mapk, phosphor-p38 Mapk, and Cyclin D1 (Cell Signaling, Boston, MA, USA) and AR and SV-40 T antigen (Santa Cruz Biotechnology). Equal protein loading was ascertained by Western blotting with β-actin antibody (Sigma-Aldrich). The intensity of each band was measured using the Image J software program, ver. 1.46 (National Cancer Institute Bethesda, MD, USA).

### Immunohistochemistry

Deparaffinised sections were incubated with antibodies for AR (Santa Cruz Biotechnology), SV40 T antigen (Santa Cruz Biotechnology), and Ki-67 (Novocastra Laboratories Ltd., Newcastle, UK). Apoptotic cells were detected by a terminal deoxy nucleotidyl transferase-mediated dUTP nick end labeling (TUNEL) assay. The TUNEL assay was performed using an *in situ* Apoptosis Detection Kit from Takara Bio, Inc. (Otsu, Japan). The labeling indices of Ki-67 and TUNEL were determined by counting at least 1,000 HG-PIN cells under a microscope at high magnification. The percentages of positive signal for AR and SV40 T antigen from total acini in each prostate lobes were acquired using an image analyzer (fluorescence microscope, BZ-9000; Keyence, Osaka, Japan). The percentage of immunopositive area in epithelial contents of acinic area was quantified by an optional software program (the BZ-analysis application; Keyence). All immunohistochemical analyses were blindly assessed by three pathologist (AN, HK and SS)

### RNA extraction and the quantitative reverse transcription-PCR (qRT-PCR) analysis

Total RNA was isolated from the lateral prostate tissues en bloc by phenol-chloroform extraction (ISOGEN; Nippon Gene Co. Ltd., Tokyo, Japan). One microgram of RNA was converted to cDNA with avian myoblastosis virus reverse transcriptase (Takara Bio, Inc.) in a 20-µl reaction mixture. Aliquots of 2 µl of cDNA samples were subjected to quantitative PCR in 25 µl using SYBR Premix ExTaq II (Takara Bio, Inc.) in a light Cycler apparatus (Roche Diagnostic, Mannheim, Germany) with universal cycling conditions. The comparative threshold cycle (C_T_) method was used to quantify data, using Gapdh as the normalizing gene. The primers used for amplification of each mRNA were as follows: rat AR forward (5′-AAT GTC CTG GAA GCC ATT GAG-3′), rat AR reverse (5′-GGA GCC ATC CAA ACT CTT GAG-3′), rat AT1R forward (5′-CAC TTT CCT GGA TGT GCT GA-3′), rat AT1R reverse (5′-CAG TGT GCT TTG AAC CTG TC-3′), rat AT2R forward (5′-ACG TGC ATG AGT GTT GAT AG-3′), rat AT2R reverse (5′-CCA TAA TAC AAG CAT TCA CAC C-3′), rat Gapdh forward (5′-GCA TCC TGC ACC ACC AAC TG-3′), and rat Gapdh reverse (5′-GCC TGC TTC ACC ACC TTG TT-3′).

The expression of AR responsive genes was determined by the Rat Androgen Receptor Signaling Targets PCR Array (Qiagen, Hilden, Germany), which allows for the simultaneous profiling of 84 androgen-regulated genes and one housekeeping gene, Actb. RNA from all rats of the control group (*n* = 12) or 2 mg/kg/day C21 group (*n* = 12) was equally mixed, and 1 µg of the RNA sample from each group was used for RT-PCR. The C_T_ method was used to quantify data using Actb as the normalizing gene. Data were analyzed using the RT^2^ Profiler PCR Array Data Analysis v3.5 from Qiagen.

### PSA measurement

LNCaP cells were cultured in Phenol Red-free RPMI1640 with10% Steroid-free FBS for 24 h. The media was changed to RPMI1640 Phenol Red-free. After a further 24 h, C21 (1 or 10 µM) with or without DHT 1 nM was added to the media. After 24 h, the media were collected to measure PSA by a chemiluminescent immunoassay (SRL Co., Tokyo, Japan).

### Statistical analysis

Differences in the quantitative data, expressed as the mean ± standard deviation (SD), between groups were compared by a one-way analysis of variance and Dunnett’s post-hoc test using the GraphPad Prism 5 software program (GraphPad Software, Inc., La Jolla, CA, USA). A *p*-value < 0.05 was considered significant.

## SUPPLEMENTARY MATERIALS FIGURES AND TABLES


