# Supplementary material for: Chemopreventive effects of angiotensin II receptor type 2 agonist on prostate carcinogenesis by the down-regulation of the androgen receptor
Source: Oncotarget. 2018 Feb 14;9(17):13859–69. doi: 10.18632/oncotarget.24492 (PMC5862621; doi:10.18632/oncotarget.24492)
Supplement: Supplementary file 1 [file oncotarget-09-13859-s001.pdf]

Chemopreventive effects of angiotensin II receptor type 2 agonist on prostate carcinogenesis by the down-regulation of the androgen receptor

SUPPLEMENTARY MATERIALS

A

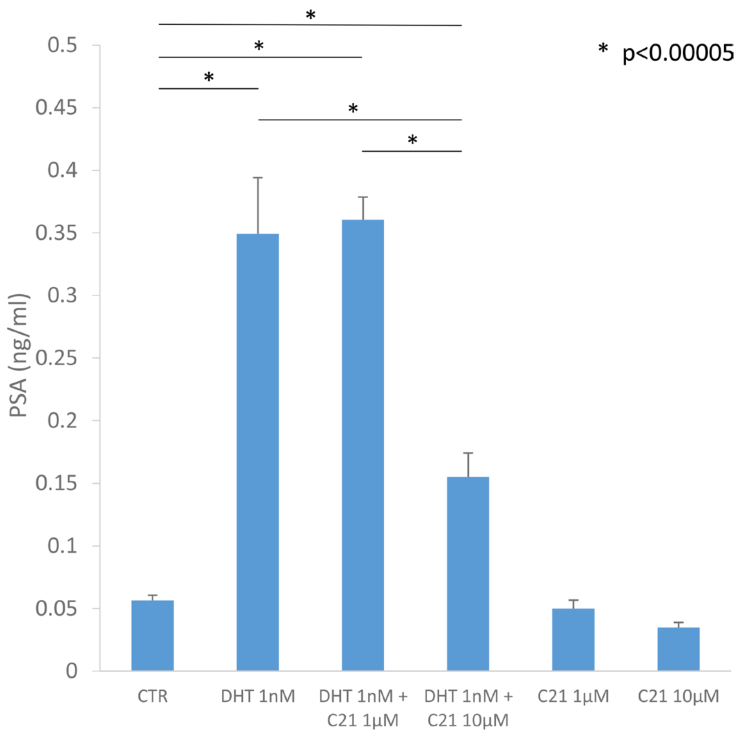

Supplementary Figure 1: C21 reduced the PSA level in the media of LNCaP cells. After the stimulation of C21 with or without DHT, the PSA levels decreased in a C21. PSA measurement was performed in LNCaP cells treated with 1 or 10 µM of C21 with or without 1 nM of DHT. Data are presented as means ± SD, $n = 6$ per group, $^{*}P < 0.00005$ vs. the control group.

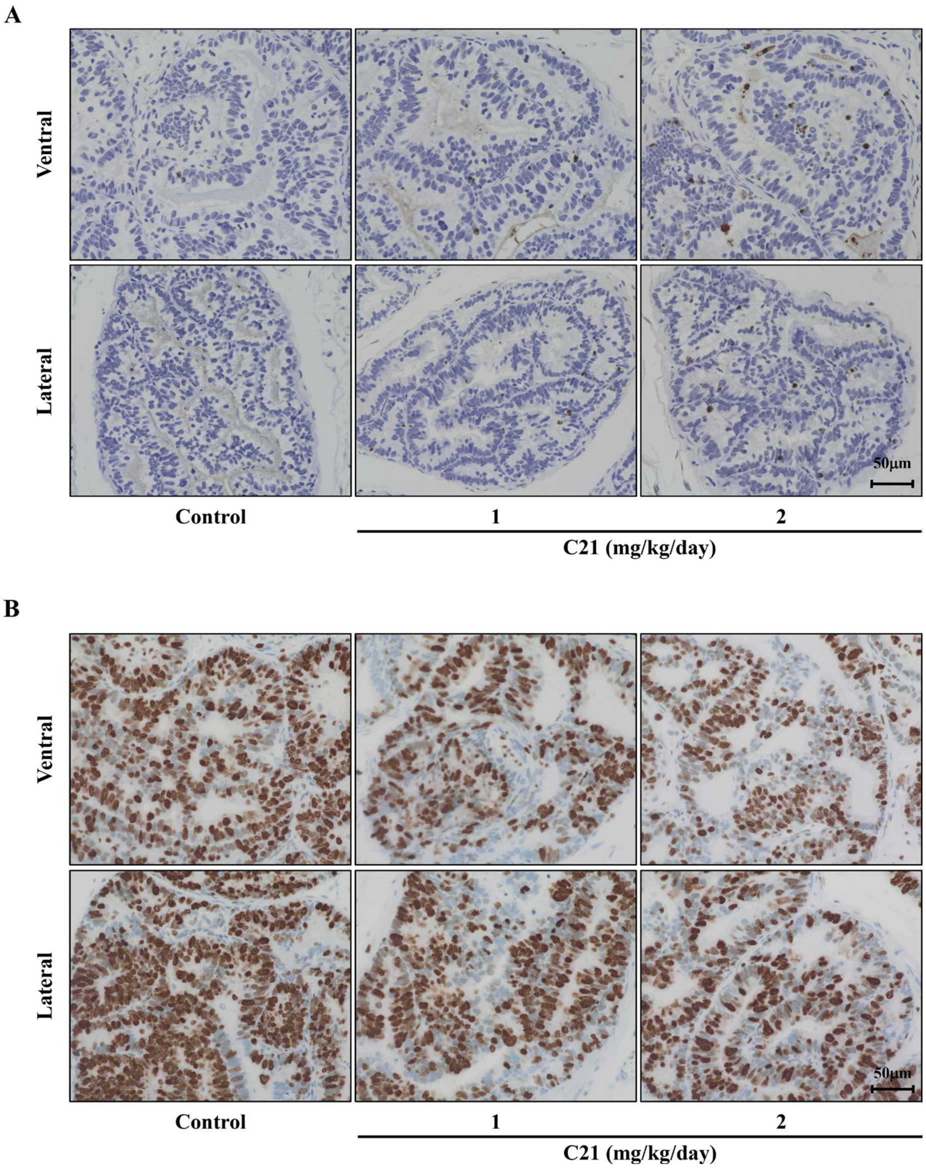

Supplementary Figure 2: AT2 agonist induced apoptosis in VP and LP of TRAP. (**A**, **B**) Immunohistochemistry of TUNEL (A) and Ki-67 (B) in HG-PIN lesions of TRAP. Hematoxylin was used as a nuclear counterstain.

Supplementary Table 1: Serum hormone levels and blood pressure of TRAP rats treated C21

	No. of rats	Testosterone (ng/ml)	Estradiol (pg/ml)	T/E2 ratio	Blood pressure (mmHg)		
					Week 0	Week 4	Week 6
Control	12	7.62 ± 2.56	17.05 ± 2.55	469 ± 199	117 ± 11	135 ± 3	133 ± 3
C21 1 mg/kg/day	12	5.46 ± 3.31	17.71 ± 3.23	319 ± 215	113 ± 12	118 ± 9**	120 ± 5**
C21 2 mg/kg/day	12	6.72 ± 3.60	19.90 ± 3.00*	330 ± 174	117 ± 6	117 ± 6***	111 ± 7****

$^{*}p < 0.05$, $^{**}p < 0.01$, $^{***}p < 0.001$, $^{****}p < 0.0001$: significantly different from control group.

Supplementary Table 2: Body and organ weights of TRAP rats treated C21

	Body weight (g)	Liver (g)	Kidney (g)	Heart (g)	Ventral prostate (g)
Control	619.7 ± 68.7	19.2 ± 2.9	3.5 ± 0.4	1.5 ± 0.2	0.28 ± 0.06
C21 1 mg/kg/day	614.7 ± 58.6	18.7 ± 2.2	3.4 ± 0.3	1.5 ± 0.1	0.27 ± 0.05
C21 2 mg/kg/day	605.9 ± 37.0	19.1 ± 1.3	3.4 ± 0.3	1.5 ± 0.1	0.28 ± 0.06
